# Prognostic nomogram for inpatients with asthma exacerbation

**DOI:** 10.1186/s12890-017-0450-2

**Published:** 2017-08-04

**Authors:** Wakae Hasegawa, Yasuhiro Yamauchi, Hideo Yasunaga, Hideyuki Takeshima, Yukiyo Sakamoto, Taisuke Jo, Yusuke Sasabuchi, Hiroki Matsui, Kiyohide Fushimi, Takahide Nagase

**Affiliations:** 1 Department of Respiratory Medicine, Graduate School of Medicine, The University of Tokyo, 7-3-1 Hongo, Bunkyo-ku, Tokyo, 113-8655 Japan; 2 Department of Clinical Epidemiology and Health Economics, School of Public Health, Tokyo, Japan; 3 Department of Health Services Research, Graduate School of Medicine, The University of Tokyo, Tokyo, Japan; 4 Department of Health Policy and Informatics, Tokyo Medical and Dental University Graduate School of Medicine, Tokyo, Japan

## Abstract

**Background:**

Asthma exacerbation may require a visit to the emergency room as well as hospitalization and can occasionally be fatal. However, there is limited information about the prognostic factors for asthma exacerbation requiring hospitalization, and no methods are available to predict an inpatient’s prognosis. We investigated the clinical features and factors affecting in-hospital mortality of patients with asthma exacerbation and generated a nomogram to predict in-hospital death using a national inpatient database in Japan.

**Methods:**

We retrospectively collected data concerning hospitalization of adult patients with asthma exacerbation between July 2010 and March 2013 using the Japanese Diagnosis Procedure Combination database. We recorded patient characteristics and performed Cox proportional hazards regression analysis to assess the factors associated with all-cause in-hospital mortality. Then, we constructed a nomogram to predict in-hospital death.

**Results:**

A total of 19,684 patients with asthma exacerbation were identified; their mean age was 58.8 years (standard deviation, 19.7 years) and median length of hospital stay was 8 days (interquartile range, 5–12 days). Among study patients, 118 died in the hospital (0.6%). Factors associated with higher in-hospital mortality included older age, male sex, reduced level of consciousness, pneumonia, and heart failure. A nomogram was generated to predict the in-hospital death based on the existence of seven variables at admission. The nomogram allowed us to estimate the probability of in-hospital death, and the calibration plot based on these results was well fitted to predict the in-hospital prognosis.

**Conclusion:**

Our nomogram allows physicians to predict individual risk of in-hospital death in patients with asthma exacerbation.

## Background

Asthma is characterized by recurrent respiratory symptoms such as wheeze, shortness of breath, chest tightness, and cough. The intensity of these symptoms varies over time together with the variable expiratory airflow limitation [1]. The treatment of asthma patients in the primary care or an outpatient setting mostly involves inhalation of corticosteroids [2]. However, since a severe exacerbation is often fatal, patients with asthma exacerbation may need to visit the emergency room followed by hospitalization and mechanical ventilation [3–6].

Although asthma mortality in adults has been decreasing since the mid-1990s [7, 8], asthma is still associated with 12 deaths per million individuals in Japan, as per data from 2014 [9]. The findings of several studies have suggested that a short-term mortality is associated with patients’ disease and drug history, including frequent use of rescue medication [10–12], less use of corticosteroids inhalation [2, 13], frequent asthma attacks, and hospitalization for asthma and impaired lung function [14, 15]. Nonetheless, there is limited understanding of the prognostic factors for patients with asthma exacerbation requiring hospitalization.

A nomogram is a graphical representation of a multivariable model that is often used for the prognosis evaluation in oncology [16, 17]. It enables physicians to generate an individual probability of clinical events, such as mortality, by integrating diverse prognostic and determinant variables [17]. With a nomogram, physicians can estimate a patient’s individual risk of a specific event at the bedside, which can be useful for clinical decision-making.

In this study, we aimed to investigate the relationships between patient characteristics and comorbidities at admission as well as the in-hospital mortality of patients with asthma exacerbation using a national inpatient database in Japan. Further, we generated a nomogram to predict the prognosis in patients with asthma exacerbation who required hospital admission.

## Methods

### Diagnosis procedure combination (DPC) database

We investigated the data abstracted from the DPC database, which is a nationwide inpatient database in Japan. The database contains administrative claims data and discharge abstract data. It mainly includes the main diagnoses, comorbidities at admission, and complications occurring during hospitalization that were diagnosed and recorded by the attending physicians and these are indicated in the database using the International Classification of Disease and Related Health Problems, 10th Revision (ICD-10) codes accompanied with text data in Japanese. The database also contains the following patient details: admission status including age, sex, height, and weight; levels of consciousness based on the Japan Coma Scale [18, 19]; levels of dyspnea based on Hugh–Jones classification [20, 21]; discharge status including in-hospital death; and medication used during hospitalization. Japan Coma Scale is defined as follows: 0, alert; I, dull; II, somnolence; and III, coma [18, 19]. The Hugh–Jones classification, which is similar to the Medical Research Council dyspnea scale and widely used in Japan, is defined as follows: I, patient’s breathing is as good as that of others with same age and build during work, walking, and climbing hills or stairs; II, patient is able to walk at pace with healthy people of same age and build on the ground level but is unable to keep up on hills or stairs; III, patient is unable to keep up with healthy people on the ground level but is able to walk about a mile or more at their own speed; IV, patient is unable to walk more than 50 yards on the ground level without taking rest; V, patient is breathless while talking or undressing or unable to leave his/her house because of breathlessness; Unspecified, patient cannot to be classified into the above grades as he/she is bedridden [20, 21].

This study was approved by the Institutional Review Board of The University of Tokyo, which waived the requirement for patient informed consent because of the anonymous nature of the data.

### Patient selection and data

We retrospectively collected the data from patients who were admitted due to asthma exacerbation and discharged between 1 July 2010 and 31 March 2013. We included patients aged ≥18 years who were diagnosed with asthma exacerbation (ICD-10 code, J45 asthma or J46 status asthmaticus as the main diagnosis, or the disease that required hospitalization) at admission and received systemic corticosteroid therapy within 2 days of admission. The exclusion criteria were as follows: patients who were transferred to other hospitals within 2 days of admission; those whose data on conscious level, dyspnea level, or body mass index (BMI) were missing; and patients with malignant diseases (C00–97), diffuse panbronchiolitis or bronchiolitis obliterans (J448), bronchiectasis (J47), tracheal or pulmonary tuberculosis (A15–16), acute ischemic heart diseases (I20–24), acute pulmonary embolism (I26), acute aortic aneurysm or dissection (I71), or acute cerebral apoplexy (I60–63) at admission. We also excluded the patients who died within 2 days of admission, as endotracheal intubation within 2 days of admission was adopted as a predictor of in-hospital death.

We identified patient characteristics at admission, including age, sex, BMI, consciousness level based on the Japan Coma Scale [18, 19], dyspnea level based on the Hugh–Jones classification [20, 21], ambulance service use, past intubation, seasonality, and requirement for intubation within 2 days of admission. The term “ambulance service use” refers to the patients who were transported to the hospital by ambulance. The term “past intubation” refers to the patients who were intubated before the index hospitalization. Seasonality included spring (March to May), summer (June to August), fall (September to November), and winter (December to February). The comorbidities that were identified using ICD-10 codes and text data in Japanese include chronic obstructive pulmonary disease (COPD, J44), pneumonia (bacterial pneumonia: J13–15, J170; atypical pneumonia: A481, J157, J160; aspiration pneumonia: J69; pneumocystis pneumonia: B59; and eosinophilic pneumonia: J82), interstitial pneumonia (J841, J848–9), heart failure (I50), chronic cerebrovascular disease (I69), chronic liver disease (K70–77), gastroesophageal reflux disease (GERD, K21), and chronic renal failure (N17–19). Other extracted data were patient outcome, including the length of hospital stay and all-cause in-hospital death.

### Statistical analysis

The chi-squared test was used to compare proportions between groups. When data were sparse (expected value <5), we used Fisher’s exact test instead of the chi-squared test to compare proportions between the groups. Cox proportional hazards regression analysis was undertaken to assess the factors associated with all-cause in-hospital mortality. The variables significantly associated with in-hospital death in the univariate analysis were used in the Cox proportional hazards regression analysis. We then used the variables identified in Cox proportional hazards regression analysis to build a nomogram that predicted in-hospital all-cause mortality. The internal validation was performed via a bootstrap method with 1000 resamples, and the calibration plot was derived to evaluate the relationship between predicted probabilities by the nomogram and observed rates. The threshold for significance was set at *P-*value <0.05. Statistical analyses were performed using SPSS version 22.0 (IBM Corp., Armonk, NY, USA), and the nomogram was built by R version 3.1.3 (The R Foundation for Statistical Computing, Vienna, Austria).

## Results

**Fig. 1:**
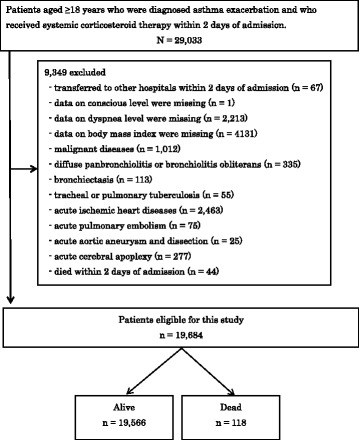
Flowchart of patient inclusion and exclusion

Among 19 million patients recorded during 33 months between July 2010 and March 2013, we identified 29,033 patients who were diagnosed with asthma exacerbation at admission and received systemic corticosteroid therapy within 2 days of admission. Among identified patients, 9349 met our exclusion criteria (Fig. 1). Ultimately, 19,684 patients were included in this study. The mean age of the patients was 58.8 years [standard deviation (SD), 19.7]. Among them, 7520 (38.2%) were male; the mean BMI was 23.7 kg/m^2^ (SD, 4.8). The median length of hospital stay was 8 days [interquartile range (IQR), 5–12 days]. Moreover, 118 patients died in the hospital (0.6%). The median length of stay for the patients who died during hospitalization was 15 days (IQR, 6.8–40.3 days).

The data represented in Table 1 show that approximately 30% (5361/19,684) of the patients used ambulance service. In-hospital mortality was significantly related to the following characteristics of patients at the time of admission: older age, male sex, disturbance of consciousness, severe dyspnea, and ambulance service use.

**Table 1:** Characteristics of inpatients with asthma exacerbation and all-cause in-hospital mortality

	Total (%)	Mortality (%)	*P*-value
Age (years)			<0.001
18–39	4136 (21.0)	1 (0.0)	
40–69	8440 (42.9)	21 (0.3)	
70–79	3857 (19.6)	30 (0.8)	
≥ 80	3251 (16.5)	66 (2.0)	
Sex			0.038
Male	7520 (38.2)	56 (0.7)	
Female	12,164 (61.8)	62 (0.5)	
BMI (kg/m^2^)			0.538
< 18.5	2135 (10.9)	18 (0.8)	
18.5–22.9	7743 (39.3)	47 (0.6)	
23.0–24.9	3316 (16.9)	19 (0.6)	
25.0–29.9	4459 (22.7)	25 (0.6)	
> 30.0	2031 (10.3)	9 (0.4)	
Consciousness level			<0.001
Alert	18,539 (94.2)	83 (0.5)	
Dull	864 (4.4)	18 (2.1)	
Somnolence	148 (0.8)	8 (5.4)	
Coma	133 (0.7)	9 (6.8)	
Dyspnea grade			<0.001
I	3476 (17.7)	8 (0.2)	
II	3392 (17.2)	10 (0.3)	
III	2971 (15.1)	9 (0.3)	
IV	4797 (24.4)	21 (0.4)	
V	5048 (25.6)	70 (1.4)	
Ambulance service use			0.024
No	14,323 (72.8)	75 (0.5)	
Yes	5361 (27.2)	43 (0.8)	
Past intubation			0.137
No	19,576 (99.5)	116 (0.6)	
Yes	108 (0.5)	2 (1.9)	
Season of admission			0.563
Spring	4225 (21.5)	25 (0.6)	
Summer	4280 (21.7)	26 (0.6)	
Autumn	5476 (27.8)	27 (0.5)	
Winter	5703 (29.0)	40 (0.7)	

*Abbreviations*: *BMI* Body mass index

The data represented in Table 2 show that common comorbidities were pneumonia, heart failure, and GERD. In-hospital mortality was related to pneumonia and heart failure.

**Table 2:** Comorbidities on admission and all-cause in-hospital mortality

	Total (%)	Mortality (%)	*P*-value
COPD			0.054
No	16,751 (85.1)	93 (0.6)	
Yes	2933 (14.9)	25 (0.9)	
Pneumonia			<0.001
No	16,960 (86.2)	81 (0.5)	
Yes	2724 (13.8)	37 (1.4)	
Interstitial pneumonia			0.344
No	19,614 (99.6)	117 (0.6)	
Yes	70 (0.4)	1 (1.4)	
Heart failure			<0.001
No	17,590 (89.4)	70 (0.4)	
Yes	2094 (10.6)	48 (2.3)	
Chronic cerebrovascular disease			0.301
No	19,293 (98.0)	114 (0.6)	
Yes	391 (2.0)	4 (1.0)	
Chronic liver disease			0.333
No	19,486 (99.0)	116 (0.6)	
Yes	198 (1.0)	2 (1.0)	
GERD			0.811
No	18,132 (92.1)	108 (0.6)	
Yes	1552 (7.9)	10 (0.6)	
Chronic renal failure			0.387
No	19,461 (98.9)	116 (0.6)	
Yes	223 (1.1)	2 (0.9)	

*Abbreviations*: *COPD* Chronic Obstructive Pulmonary Disease, *GERD* Gastroesophageal reflux diseases

The number of patients who required mechanical ventilation during hospitalization was 769 (3.9%). Among them, 85% patients (*n* = 649; 3.3%) required mechanical ventilation within 2 days of admission (Table 3). The median time from admission to intubation was 0 days (IQR, 0–1 day), and the median length of ventilation period was 2 days (IQR, 1–5 days). Further, 12.1% of patients who underwent intubation (*n* = 108; 0.5%) required one or more further episodes of mechanical ventilation during the study period. The data depicted in Table 3 also shows that in-hospital mortality was related to endotracheal intubation within 2 days of admission. However, a total of 24 patients (3.7%) died after mechanical ventilation.

**Table 3:** Clinical course after admission and all-cause in-hospital mortality

	Total (%)	Mortality (%)	*P*-value
Intubation within two days of admission			<0.001
No	19,035 (96.7)	94 (0.5)	
Yes	649 (3.3)	24 (3.7)	

The data represented in Table 4 shows the Cox proportional hazards regression analysis for all-cause in-hospital mortality. Although ambulance service use was significantly correlated with in-hospital death in the univariate analysis, it was not included in the Cox proportional hazards regression analysis as it is influenced by the patient’s background. According to Cox proportional hazards regression analysis, higher mortality among patients with asthma exacerbation was associated with older age, male sex, disturbance of consciousness, pneumonia, and heart failure at admission.

**Table 4:** Cox proportional hazards regression analysis for all-cause in-hospital mortality

		Hazard ratio	95% confidence interval	*P*-value
Age (years)	18–39	Reference		
	40–69	7.46	1.00–55.63	0.050
	70–79	12.13	1.64–89.87	0.015
	≥80	21.38	2.92–156.46	0.003
Sex (Female)		0.61	0.42–0.88	0.008
Consciousness level	Alert	Reference		
	Dull	1.89	1.11–3.21	0.018
	Somnolence	5.39	2.43–11.96	<0.001
	Coma	9.68	4.18–22.41	<0.001
Dyspnea grade	I	Reference		
	II	0.79	0.31–2.02	0.625
	III	0.64	0.24–1.66	0.354
	IV	0.67	0.29–1.54	0.345
	V	1.51	0.71–3.20	0.284
Intubation within two days of admission		1.35	0.76–2.40	0.306
Pneumonia		1.81	1.21–2.69	0.004
Heart failure		2.08	1.41–3.06	<0.001

**Fig. 2:**
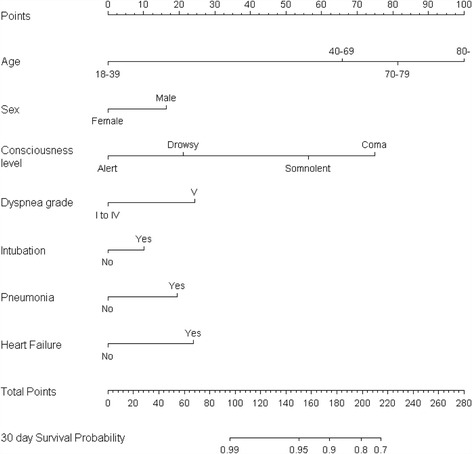
A nomogram to predict in-hospital mortality in patients with asthma exacerbation. The patient’s status for each predictor is plotted on the horizontal scale as axis points, and the vertical lines are drawn up to the axis points to obtain the corresponding points. After all points are summed, the total point score on the total point line is plotted and a vertical line is drawn down to the bottom line. The corresponding value shows the predicted probability of in-hospital death (for example, an 80-year-old (100 points) alert woman with pneumonia on admission (20 points) along with grade I dyspnea without any evidence of heart failure and requirement for tracheal intubation would score 120 points. Her 30-day survival probability is 0.95–0.99). Level of consciousness was estimated using the Japan Coma Scale, and dyspnea was estimated with Hugh–Jones classification. The term “intubation” refers to the intubation within 2 days of hospitalization

**Fig. 3:**
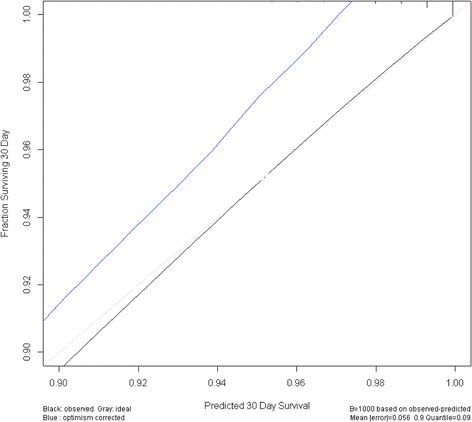
Calibration plot. The gray line at 45° indicates the ideal nomogram reference line. The black line shows the calculated data from the dataset. The optimism corrected line is an adjusted line generated by a bootstrap method with 1000 resamples

The nomogram was built using the same variables identified in the Cox proportional hazards regression analysis (Fig. 2). It allowed us to estimate the probability of in-hospital death. The concordance index of the nomogram was 0.869 (95% confidence interval, 0.868–0.869). Internal validation was performed by a bootstrap method with 1000 resamples. The calibration plots are shown in Fig. 3.

## Discussion

We investigated the clinical characteristics, admission status, and all-cause in-hospital mortality of patients with asthma exacerbation who required hospitalization. We also developed a nomogram that predicted in-hospital mortality. Our study revealed that the mortality rate of patients who were admitted due to exacerbation was 0.6%. Moreover, in-hospital mortality was associated with older age, male sex, disturbance of consciousness, pneumonia, and heart failure at admission. A nomogram was generated based on seven variables at admission to predict in-hospital death.

In this study, the in-hospital mortality rate for asthma was 0.6%, which is broadly comparable to the previous reports based on nationally representative data of the US (0.5%) [5] and UK (0.43%) [7]. There may be several reasons behind the difference in asthma-related mortality observed in our study with that observed in previous studies; one of the most likely reasons may be the age difference among patients included in the study. Our study did not include young patients, including infants and children, and the proportion of older patients was higher than that in previous reports. Since young patients with asthma are reported to have relatively favorable outcomes [22–24], it is likely that in-hospital mortality is higher in the present study than previous reports. The proportion of patients who required intubation/mechanical ventilation was 3.9%, which is similar to that indicated by the previous US report (4.2%) [5].

Patients with clinical features of both asthma and COPD have been referred to have “asthma COPD overlap” (ACO) syndrome [25]. Our previous study has demonstrated that compared with asthma alone, patients with ACO exhibit significantly higher in-hospital mortality [26]. In this study, although the in-hospital mortality of patients with ACO was higher than that of patients with asthma alone, the difference was not significant, probably because of the low death rate among the study population.

Previous studies have reported that short-term mortality of patients with asthma exacerbation is associated with older age [5, 23, 24, 27], male sex [5, 27], transferred on admission [5], mechanical ventilation [5, 27], past intubation [27–29], and more comorbid conditions [5, 27, 29]. Our study also identified these factors as the predictors of short-term in-hospital mortality in patients with asthma exacerbation. To the best of our knowledge, the present study reveals for the first time that in-hospital mortality is associated with lower level of consciousness.

Hypercapnia, which is defined as higher partial pressure of arterial CO_2_, is known to be associated with severe airway obstruction in patients with asthma and may impair the level of consciousness [30] and ability to talk or move. One previous study has reported no significant relationship between hypercapnia and death in outpatients with asthma [31], while another study has shown that higher partial pressure of arterial CO_2_ is associated with increased mortality in patients admitted to the intensive care unit with asthma exacerbation [32]. Thus, hypercapnia may be a potential risk factor associated with patient’s mortality in extreme conditions. In this context, our study, which included such patients, may explain the association between impaired consciousness and asthma mortality in adults.

Our nomogram enables to predict a patient’s risk of in-hospital death by evaluating simple parameters such as personal characteristics, medical history, and physical status at admission, without the need for complex examinations or investigations. One of the advantages of a nomogram is its ability to estimate individual risk in a simple and straightforward manner [17]. Nomograms are widely used as the prognostic devices in oncology [16, 17]. In respiratory medicine, nomograms have been used to predict survival in patients with non-small cell lung cancer [33] or probability of asthma diagnosis [34]; however, we are the first to build a nomogram that predicts in-hospital mortality in patients with asthma exacerbation. Previous studies that have identified the risk factors associated with death in patients with asthma fail to identify the means of predicting the probability of death in individual patients. A nomogram can integrate the variables and relevant determinants of a disease into a prognosis [17]. With the present nomogram, physicians can easily estimate a patient’s individual probability of death, which is helpful for clinical decision-making.

However, our study has certain limitations, one of which is the accuracy of asthma diagnosis. Because this study was a retrospective investigation based on information from an administrative database with de-identified data, we were unable to confirm how the patients were diagnosed with asthma. However, the diagnoses were recorded by the attending physicians, and we selected the patients who received systemic corticosteroid therapy for acute asthma attack soon after admission. Thus, we believe that the accuracy of the diagnosis in our study is adequate. Further, because the DPC database does not include detailed clinical information such as that regarding drugs used before or after admission, we could not evaluate the results of pulmonary function or blood tests, socioeconomic variables, symptoms before admission, and the baseline status of patients. Finally, because of missing data, we could not investigate the influence of smoking history on the outcome of asthma exacerbation.

## Conclusions

All-cause in-hospital mortality in patients with asthma exacerbation is associated with older age, male sex, lower level of consciousness, pneumonia, and heart failure at admission. Our nomogram is a simple and straightforward means of helping physicians to predict individual risk of in-hospital death in patients with asthma exacerbation.

## Abbreviations

ACO: And asthma COPD overlap
BMI: Body mass index
COPD: Chronic obstructive pulmonary disease
DPC: Diagnosis Procedure Combination
GERD: Gastroesophageal reflux disease
ICD-10: International Classification of Disease and Related Health Problems, 10th Revision
IQR: Interquartile range
SD: Standard deviation
